# Sweetness and light: illuminating the honey bee genome

**DOI:** 10.1111/j.1365-2583.2006.00698.x

**Published:** 2006-10

**Authors:** G E Robinson, J D Evans, R Maleszka, H M Robertson, D B Weaver, K Worley, R A Gibbs, G M Weinstock

**Affiliations:** Department of Entomology, University of Illinois at Urbana-Champaign Urbana, IL, USA; * Bee Research Laboratory, USDA-ARS, BARC-E Beltsville, MD, USA; † Visual Sciences & ARC Centre for Molecular Genetics of Development, Research School of Biological Sciences, The Australian National University Canberra, Australia; ‡ Bee Power, L.P. Navasota, TX, USA; § Human Genome Sequencing Center, Baylor College of Medicine Houston, TX, USA

Instead of dirt and poison we have rather chosen to fill our hives with honey and wax; thus furnishing mankind with two of the noblest things, which are sweetness and light.

Jonathan Swift

The honey bee *Apis mellifera* is the first hymenopteran and the fifth insect genome to be sequenced (Honey Bee Genome Sequencing Consortium, 2006) in what promises to be a swarm of insect genome sequences expected to appear over the next few years (Table 1). The Honey Bee Genome Sequencing Project (HBGSP) was conceptualized over a period from 1998 to 2001 by the community at courses, conferences and workshops (Robinson, 1999; Maleszka, 2000; Pennisi, 2001). In addition, initial efforts were directed at physical and genetic maps of the genome (Estoup *et al*., 1995; Hunt & Page, 1995), collections of expressed sequence tags (Evans & Wheeler, 2000; Whitfield *et al*., 2002), and studies using microarrays (Kucharski & Maleszka, 2002; Takeuchi *et al*., 2002; Whitfield *et al*., 2003).

**Table 1:** Genome projects of insects and other arthropods

Organism	Common name	Order	Size (Mb)	Status	Sequencing centres*	Reference/source
*Acyrthosiphon pisum*	Pea aphid	Hemiptera	525	Ongoing	BCM-HGSC	http://www.hgsc.bcm.tmc.edu
*Aedes aegyptii*	Mosquito	Diptera	1310	Complete	BI, TIGR	http://msc.tigr.org/aedes/aedes.shtml http://www.broad.mit.edu/annotation/disease_vector/aedes_aegypti/
*Anopheles gambiae*	Mosquito	Diptera	264	Complete	Celera Genomics, Genoscope, the University of Notre Dame, EBI/Sanger Institute, EMBL, Institut Pasteur, IMBB and TIGR	Holt *et al*. (2002); Mongin *et al*. (2004)
*Apis mellifera*	Honey bee	Hymenoptera	262	Complete	BCM-HGSC	Honey Bee Genome Sequencing Consortium (2006)
*Bicyclus anynana*	Butterfly	Lepidoptera	500	Ongoing	JGI	http://www.jgi.doe.gov/sequencing/why/CSP2006/butterfly.html
*Bombyx mori*	Silkworm	Lepidoptera	530	Complete	International Lepidopteran Genome Project	Mita *et al*. (2004); Xia *et al*. (2004)
*Culex pipiens*	Mosquito	Diptera	540	Ongoing	BI, TIGR	http://msc.tigr.org/c_pipiens/index.shtml http://www.broad.mit.edu/seq/msc/
*Daphnia pulex*	Water flea	Siphonaptera	200	Ongoing	JGI	wfleabase.org
*Drosophila melanogaster*	Fruit fly	Diptera	132	Complete	CGI; BDGP; BCM-HGSC	Adams *et al*. (2000); Celniker *et al*. (2002)
*Drosophila* pseudoobscura	Fruit fly	Diptera	139	Complete	BCM-HGSC	Richards *et al*. (2005)
*Drosophila* species†	Fruit fly	Diptera	∼135	Ongoing	Multicentre2	flybase.bio.indiana.edu
*Glossina morsitans*	Tsetse fly	Diptera	590	Ongoing	WTSI	http://www.sanger.ac.uk/Projects/G_morsitans/
*Ixodes scapularis*	Tick	Acarina	2100	Ongoing	BI, TIGR	http://www.entm.purdue.edu/igp/default.html
*Nasonia* sp.	Wasp	Hymenoptera	345	Ongoing	BCM-HGSC	http://www.hgsc.bcm.tmc.edu
*Pediculus humanus*	Body louse	Phthiraptera	107	Ongoing	JCVI	http://www.entm.purdue.edu/pittendrigh_lab/default.html
*Rhodnius prolixus*	Chagas’ disease vector	Hemiptera	670	Ongoing	WUGSC	http://www.genome.wustl.edu/genome.cgi?GENOME=Rhodnius%20prolixus
Sand flies‡	Sand fly	Diptera	170–300	Ongoing	BCM-HGSC, WUGSC	http://www.genome.wustl.eduhttp://www.hgsc.bcm.tmc.edu
*Tribolium castaneum*	Red flour beetle	Coleoptera	158	Ongoing	BCM-HGSC	http://www.hgsc.bcm.tmc.edu/projects/tribolium/

* ABC, Agencourt Bioscience Corp.; BCM-HGSC, Baylor College of Medicine Human Genome Sequencing Center; BDGP, Berkeley *Drosophila* Genome Project; BI, Broad Institute; CGI, Celera Genomics Inc.; JCVI, J. Craig Venter Institute; JGI, Department of Energy Joint Genome Institute; TIGR, The Institute for Genomic Research; WTSI, Wellcome Trust Sanger Institute; WUGSC, Washington University Genome Sequencing Center.

† *Drosophila* species being analysed and the centres performing this work are *virilis*, *ananassae*, *mojavensis*, *erecta*, *grimshawi* (ABC); *willistoni* (JCVI); *persimilis* and *sechellia* (BI); *yakuba* and *simulans* (WUGSC).

‡ *Lutzomiya longipalpis* and *Phlebotomus papatasi*, EST sequencing.

At the end of 2001 members of the honey bee community, led by Gene Robinson and Daniel Weaver, and the United States Department of Agriculture, represented by Kevin Hackett, met at the Baylor College of Medicine Human Genome Sequencing Center (BCM-HGSC) to discuss a full genome sequencing project. (Representatives of the bovine community were also at this meeting to discuss their genome project, a gathering warmly remembered as the milk and honey workshop.) A White Paper to the National Human Genome Research Institute of the NIH ensued (Honey Bee Genome Sequencing Consortium, 2002), which led to the HBGSP receiving a high priority ranking in the comparative genomics program at the NHGRI. With this support from NHGRI, and additional contributions from the USDA resulting from the efforts of Under Secretary Joseph Jen, the project began in December 2002 at BCM-HGSC.

All genome projects have their challenges as each genome and organism has its own idiosyncrasies. The honey bee was no different. A principal complication was under-representation of AT-rich regions of the genome among the small insert shotgun libraries constructed in *Escherichia coli* for the bulk of the sequencing. Possibly AT-rich DNA was degraded during the preparation of libraries or the clone inserts were not maintained in *E. coli*. To overcome this, Martin Beye supplied AT-rich DNA isolated from dye-CsCl gradients, and this was used to make more shotgun libraries to build up coverage of the AT-rich regions. It was also found that the genome was not fully represented in the large insert BAC clone library, which again could reflect either loss of some regions during clone preparation or in *E. coli*. The BAC problem was never solved and so these clones were used sparingly in the project. A potential problem, polymorphism making it difficult to assemble shotgun sequences, was managed using a partially inbred queen from Daniel Weaver. The DNA for sequencing came from a large number of drones. Although polymorphism was not insignificant, several polymorphic alleles per kilobase, this was a boon for identifying SNPs and quite manageable in genome assembly.

The lack of BAC clones meant that the HBGSP became a pure Whole Genome Shotgun project. In all, the project produced over three million DNA sequences for assembly, mainly from small insert clones, but including a few fosmid and BAC clones. The genome assembly used over 80% of these data. The reads were assembled into the genome with the Atlas assembly software, developed at the BCM-HGSC (Havlak *et al*., 2004). All overlaps between reads were first found by an alignment process and highly repeated sequences were identified because of their large number of overlapping reads. These were set aside, and then a series of steps were performed to create a layout of the reads based on their overlapping sequences. This resulted in clusters of overlapping reads (bins of reads), which end in gaps where the repeated sequences have been removed. Each bin of reads was then assembled into a consensus sequence using Phrap (Ewing & Green, 1998; Ewing *et al*., 1998), generally producing a single contig (a continuous stretch of sequence). Contigs were linked together into scaffolds using the read pairing information (each clone is sequenced from both ends, producing a pair of reads). The highly repeated sequences were now added back to the assembly, using the read pair information for their placement. The scaffolds were used to build chromosomes, by aligning them to the markers of the linkage map (Solignac *et al*., 2003, 2004, 2006), called superscaffolding. Manual superscaffolding was also performed by placing reads that were not used by these automated procedures.

The product of these activities was a draft assembly, a consensus sequence good enough to represent nearly all genes at a quality sufficient for use in searches (e.g. with Blast). There are gaps, mainly due to repeats that could not be unambiguously placed, which are of lesser interest than transcribed regions. There are low coverage regions, mainly due to AT cloning bias, but there is enough coverage to find genes in these regions. Some of the assembly was not placed on chromosomes: these tend to be short contigs that fall between markers, especially where markers are far apart. Efforts were made during the project to systematically find markers to fill in these holes so this problem was minimized.

In addition to the draft assembly, a collection of single nucleotide polymorphisms was produced as part of the project. Although the queen used was partially inbred, considerable polymorphism was present among the scores of pooled drones used as DNA sources. Analysis of these sequences at the BCM-HGSC resulted in identification of about 1 million candidate SNPs. Likewise, DNA was prepared and sequenced from Africanized honey bees and these individual sequences were compared with the assembled honey bee sequence to identify more SNPs. Both of these data sets have been submitted to dbSNP. Whitfield *et al*. (Honey Bee Genome Sequencing Consortium, 2006; Whitfield *et al*., 2006) performed similar SNP discovery efforts with these Africanized sequences as well as ESTs.

The gene list produced from the honey bee genome sequence was generated via a novel method. Five different gene lists were merged using the GLEAN program (Liu *et al*., 2006) to produce a consensus set that was superior to any of the individual lists (Elsik *et al*., 2006). In addition an *ab initio* list, from Fgenesh (Salamov & Solovyev, 2000), a gene prediction program that overcalls possible genes, was used. The GLEAN and *ab initio* gene lists were tested against a genome-wide oligonucleotide array (HBGSC, 2006), another first for insect projects. These efforts produced a list of about 10 000 genes, fewer than predicted in other insect projects. The high quality *Drosophila melanogaster* genome has about 13 000 predicted genes, while higher numbers are predicted for *Anopheles* and *Bombyx*. These latter genomes may be overestimates due to redundancy and polymorphism in the assemblies, while the *Drosophila* number is likely very accurate. Why is the *Apis* number so low? We believe this is mainly due to lack of EST and cDNA evidence and a conservative gene calling approach. We expect this number to increase in the future.

What are the limitations of this current low number for the honey bee gene list? We expect the deficit to be mainly in unique genes or rapidly evolving genes that are hard to identify by comparison with other genomes. In contrast, we expect gene families, which are primarily the subject of the analyses presented in the papers in this special issue, to be more completely represented. However, this is the nature of a draft genome and it provides defined measures for future upgrading.

Genome analysis was performed with maximum community engagement. The HBGSP united a broad range of scientists, from leaders in human genomics and bioinformatics at BCM-HGSC and elsewhere to members of diverse disciplinary and organism-based communities, including those studying mammals and humans. A total of 112 individuals in 63 institutions around the world signed on to analyse the newly available honey bee genome sequence, generating exciting results in many areas of biology. Themes for analysis were identified by the HBGSP and analysis teams for each of these areas were formed. The analysis themes included Anti-xenobiotic Defence Mechanisms, Bee Disease and Immunity, Brain and Behaviour, Caste Development and Reproduction, Comparative and Evolutionary Analysis, Development and Metabolism, Gene Regulation, Genome Analysis, Physical and Genetic Mapping and Chromosome Structure, Population Genetics, Repeated Sequences and Transposable Elements.

These groups manually analysed over 3000 gene models and identified changes in gene family numbers or in the genetic composition of pathways, by comparison with other insect genomes as well as other genomes, particularly the human genome. In addition there was considerable effort to confirm missing genes: these may be truly absent or they may be present but not recognized if they have a rapidly evolving sequence.

A principal focus was on the honey bee complex social life-style and how it differs from other solitary life-style insects. This large community effort is presented in a special issue of *Nature* (Honey Bee Genome Sequencing Consortium, 2006) and in more detail in a large number of companion papers forming this issue as well as in other journals. Papers appearing in this volume of *Insect Molecular Biology* provide new insights into diverse topics in honey bee biology, including neurobiology (Eisenhardt & Leboulle, 2006) and the process of caste determination that results in reproductive queens and largely sterile workers (Cristino *et al*., 2006; Wheeler *et al*., 2006). They also address some of the challenges faced by honey bees, including analyses of disease-resistance pathways (Evans *et al*., 2006; Zou *et al*., 2006; Claudianos *et al*., 2006) and metabolic adaptations to an all floral (pollen and nectar) diet (Kunieda *et al*., 2006). Several papers address ways that honey bee studies can provide insights into human health. These papers cover the genetic bases of honey bee venom allergens (Peiren, 2006), along with mechanistic insights into the remarkable longevity of queen honey bees (Corona & Robinson, 2006) and sperm stored in the spermatheca (Collins *et al*., 2006). All told over 50 papers will be appearing from this work.

The HBGSP has so far produced a prodigious amount of information, and online resources and database development is proceeding aggressively to manage this (Table 2). BeeBase is a dedicated analysis and display environment for the honey bee genome, headed by Christine Elsik, Texas A&M University, which will be closely tied to the famous FlyBase in collaboration with William Gelbart (Harvard University). Other databases include: NCBI Honey Bee Genomic Resource, EBI-Heidelberg, UC Santa Cruz, US-DOE, and the central site at BCM-HGSC. The BCM-HGSC site also offers the genome sequences for two key honey bee pathogens, *Paenibacillus larvae* and *Ascosphaera apis*, projects funded by USDA-ARS (Kate Aronstein and Jay Evans, Principal Investigators) and described in this special issue (Qin *et al*., 2006). BeeSpace is a project funded by NSF's Frontiers in Biological Research Program, headed by Bruce Schatz (University of Illinois at Urbana-Champaign), for information scientists and biologists to leverage the bee genome to create a new information environment for the study of social behaviour (http://www.beespace.uiuc.edu). New genomic resources are being created in collaboration with industry leaders, government labs, and academia, including whole genome microarrays (Viktor Stolc, NASA-Ames; and Gene Robinson, Jay Evans and Kevin White) and large-scale collections of SNPs for European and Africanized honey bees (above).

**Table 2:** Honey bee genome resources

Resource	Reference
Genome assemblies	ftp://ftp.hgsc.bcm.tmc.edu/pub/data/Amellifera/fasta/
NCBI version 4 assembly	Accession nos CM000054–CM000069, CH876891–CH878241
Browsers	BeeBase (racerx00.tamu.edu/bee_resources.html)
	NCBI (http://www.ncbi.nlm.nih.gov/genome/guide/bee)
	UCSC (genome.ucsc.edu).
Manual Superscaffolds for chromosomes 13, 14, 15, 16	racerx00.tamu.edu/bee_resources.html
SNPs from BCM-HGSC	dbSNP at NCBI (http://www.ncbi.nlm.nih.gov/entrez/query.fcgi?db=Snp&cmd=limits)
	BCM HGSC ftp site (ftp://ftp.hgsc.bcm.tmc.edu/pub/data/Amellifera/snp)
SNPs from ESTs	from UIUC (titan.biotec.uiuc.edu/bee/downloads/bee_downloads.html).
Tiling Array Data	http://www.systemix.org
Gene Predictions	BeeBase racerx00.tamu.edu/downloadFASTA.html

The HBGSP has produced an excellent draft honey bee genome sequence, enhanced by coordinating the assembly of the genome at BCM-HGSC and the mapping of the genome by Michel Solignac and colleagues at INRA, France (Solignac *et al*., 2003, 2004, 2006). To further increase the value of the honey bee genome sequence to researchers, a White Paper to obtain additional sequence information was submitted to NHGRI in July 2005 (Honey Bee Genome Sequencing Consortium, 2005). The project was accorded ‘High Priority’ in August 2005, and this work will begin late in 2006. The HBGSP is expected to usher in a bright era of bee research, for the benefit of agriculture, biological research and human health.
